# Biomarkers in Cardiorenal Syndromes

**DOI:** 10.1155/2018/9617363

**Published:** 2018-03-05

**Authors:** Shihui Fu, Shaopan Zhao, Ping Ye, Leiming Luo

**Affiliations:** ^1^Department of Geriatric Cardiology, Chinese People's Liberation Army General Hospital, Beijing 100853, China; ^2^Department of Cardiology and Hainan Branch, Chinese People's Liberation Army General Hospital, Beijing 100853, China

## Abstract

There is a consensus that cardiorenal syndromes (CRS) are defined as the disorders of heart and kidney where acute or chronic dysfunction in one organ may induce acute or chronic dysfunction in another. Patients with CRS have increased hospitalization and mortality rates, and thus their identification is of great implication. Biomarkers are not only predictive in heart failure or renal diseases, but also useful in identifying cardiac dysfunction in renal diseases and renal injury in heart failure. Thus, they may be applied in order to identify patients with CRS and even assess prognosis and guide therapy in these patients. However, studies on biomarkers have just begun in CRS. Future studies are essential to observe current biomarkers and find novel biomarkers in CRS so as to improve diagnosis, therapy, and prognosis of CRS.

## 1. Introduction

There is a consensus that cardiorenal syndromes (CRS) are defined as the disorders of heart and kidney where acute or chronic dysfunction in one organ may induce acute or chronic dysfunction in another [1]. They include five subtypes: (1) type 1 (acute cardiorenal syndrome), acute heart failure (AHF) leading to acute kidney injury (AKI) mediated by hemodynamic, humoral, hormonal, and immune factors; (2) type 2 (chronic cardiorenal syndrome), chronic heart failure (CHF) leading to chronic kidney disease (CKD) mediated by low cardiac output, chronic hypotension, increased venous pressure, subclinical inflammation, endothelial dysfunction, and accelerated atherosclerosis; (3) type 3 (acute renocardiac syndrome), AKI leading to AHF mediated by vasoconstriction, volume expansion, humoral signaling, electrolyte abnormalities, coagulation imbalance, activated renin-angiotensin-aldosterone system (RAAS), and sympathetic nervous system (SNS); (4) type 4 (chronic renocardiac syndrome), CKD leading to CHF mediated by anemia, malnutrition, Na-H_2_O overload, uremic toxins, Ca/P abnormalities, and chronic inflammation; (5) type 5 (secondary CRS), systemic conditions leading to simultaneous dysfunction of the heart and kidney mediated by hemodynamic changes, neurohumoral activation, and altered metabolism and immune response [1]. Patients with CRS have increased hospitalization and mortality rates, and thus their identification is of great implication [2]. Biomarkers are not only predictive in heart failure (HF) or renal diseases, but also useful in identifying cardiac dysfunction in renal diseases and renal injury in HF. Thus, they may be applied in order to identify patients with CRS and assess prognosis and guide therapy in these patients (Table 1) [1, 2].

## 2. Cardiac Biomarkers

### 2.1. Cardiac Troponin

As the components of the contractile apparatus in cardiomyocytes, cardiac troponin T (cTnT) and cardiac troponin I (cTnI) are specific biomarkers of myocardial injury and infarction [3]. cTnT and cTnI correlate with ventricular remodeling after HF and increase as HF progresses and mortality rises [4]. Abnormal hemodynamics (ventricular overload and hypertrophy) and neuroendocrine (activated RAAS and SNS) result in cardiomyocyte necrosis and apoptosis and elevated cTnT and cTnI levels in patients with HF [5]. cTnT and cTnI predict prognosis and stratify risk in patients with HF [6]. Patients with CKD have elevated cTnT and cTnI levels because of the reduced excretion from the kidney [7]. cTnT and cTnI predict cardiovascular and all-cause mortality rates in patients with mild-to-moderate CKD or end-stage renal disease (ESRD) [8]. Elevated cTnT and cTnI levels are related to myocardial injury, ventricular hypertrophy, HF, and CKD, and there are more individuals with elevated cTnT and cTnI levels due to the use of high-sensitivity assays in the general population [9]. Regardless of using a high-sensitivity assay or not, cTnT and cTnI predict hospitalization and mortality rates in the general population.

### 2.2. B-Type Natriuretic Peptide

B-type natriuretic peptide (BNP) is mainly released from cardiomyocytes and plays diuretic, natriuretic, vasodilative, and other cardioprotective roles. Patients with HF have overactivated RAAS and SNS and compensated release of BNP due to ventricular volume and pressure overload. But BNP is still deficient and its roles are resisted in these patients [10]. As two preferred biomarkers of HF, BNP and N-terminal proBNP (NT-proBNP) correlate with HF symptom [New York Heart Association (NYHA) classification], cardiac function [left ventricular ejection fraction (LVEF)], ventricular pressure, and wall thickness. BNP and NT-proBNP are elevated in patients with HF, and they help assess prognosis and stratify risk in these patients [11]. BNP and NT-proBNP have superior abilities to predict prognosis compared with traditional cardiovascular risk factors, including age, NYHA classification, ventricular dilation, and renal dysfunction, and neuroendocrine indicators, including norepinephrine, renin, angiotensin, aldosterone, and endothelin [12, 13]. BNP and NT-proBNP assess severity and assess prognosis in patients with systolic or diastolic dysfunction. Moreover, repeated BNP and NT-proBNP assays can provide more information on risk stratification. BNP and NT-proBNP predict cardiovascular events in the general population [14]. BNP has been considered to guide therapy and assess its effects, and BNP-guided therapy may reduce all-cause mortality rates in patients with HF [15]. However, the benefit of BNP-guided therapy in HF is based on relatively low-quality evidence and not completely established as a standard method. Future models based on BNP and NT-proBNP may benefit diagnosis and therapy of patients with HF [16]. Patients with renal dysfunction have reduced excretion of BNP and NT-proBNP from the kidneys. BNP and NT-proBNP correlate with renal function and predict cardiovascular and all-cause mortality rates, independent of cardiovascular diseases and their severity [17]. NT-proBNP is more sensitive to renal function than BNP [18]. Relative effects of heart and kidney on plasma BNP and NT-proBNP levels remain unclear in patients with CRS [19]. Kidneys may play a more significant role than the heart in patients with ESRD, and the heart may play a more significant role than the kidneys in other patients with CKD.

### 2.3. C-Reactive Protein

As a biomarker of systematic inflammation, C-reactive protein (CRP) may affect endothelial dysfunction through increasing expression of endothelial cell adhesion molecules, decreasing nitric oxide and prostaglandin released from endothelial cells, augmenting low-density lipoprotein uptake by macrophages, and inducing complement-mediated inflammatory reaction [20]. Moreover, CRP may affect coagulative and fibrinolytic systems and inhibit ventricular function. CRP has elevated levels and predicts prognosis in patients with cardiac or renal dysfunction [21, 22]. Compared with traditional cardiovascular risk factors, CRP has an additional ability to predict cardiovascular risk in the general population [23]. Plasma CRP levels correspond to the following cardiovascular risk: low risk (<1 mg/L), middle risk (1–3 mg/L), high risk (>3 mg/L), and extremely high risk (>10 mg/L) [24]. Plasma CRP levels >2 mg/L predict increased death risk in the general population [25]. CRP is related to both microalbuminuria and macroalbuminuria. CRP increases glomerular capillary permeability and glomerular mesangial cell hyperplasia through mediating vasoconstriction, embolism, and inflammation. CRP predicts prognosis and stratifies risk in patients with ESRD [26].

### 2.4. Myeloperoxidase

Myeloperoxidase (MPO) is produced by neutrophils and monocytes. A series of reactive oxidation substances are produced by MPO catalysis [27]. MPO is related to oxidative stress, inflammatory reaction, ventricular remodeling, vulnerable plaque, and metabolism of muscle cells [28]. MPO predicts prognosis in patients with AHF, independently of traditional cardiovascular risk factors. Moreover, MPO has elevated levels in patients with CHF and can identify patients with CHF in the general population. Regardless of systolic or diastolic HF and left or right HF, plasma MPO levels positively correlate with CHF progression and predict cardiovascular events in patients with CHF [29]. MPO also predicts CKD severity and mortality rates in patients with CKD [30].

### 2.5. Soluble Vascular Endothelial Growth Factor Receptors-1

Vascular endothelial growth factor (VEGF) promotes the growth of endothelial cells and prevents apoptosis of endothelial cells and increases nitric oxide and prostaglandin released from endothelial cells. VEGF, platelet-derived growth factor (PDGF), and soluble vascular endothelial growth factor receptors-1 (sFlt-1) have elevated levels in patients with HF. PDGF reduces infarct size and improves cardiac dysfunction, whereas sFlt-1 may inhibit these roles of PDGF. sFlt-1 is a biomarker that predicts prognosis in patients with HF [31]. VEGF may identify the development of CKD and predict mortality in patients with CKD. sFlt-1 contributes to endothelial dysfunction in CKD and links microvascular disease with HF in CKD [32]. The ratio of PDGF to sFlt-1 correlates with HF severity in patients with renal dysfunction.

### 2.6. Copeptin

Copeptin, a glycosylated 39-amino-acid peptide, is a C-terminal part of precursor pre-provasopressin (pre-proAVP) and is released in the same amount as AVP. Whether the heart also releases copeptin into the blood remains controversial [33]. AVP has a half-life of 5–20 minutes, whereas copeptin has a half-life of days [34]. Copeptin takes place of AVP as a liable biomarker of cardiovascular diseases as well as a significant predictor of mortality [35]. It has been regarded as a hallmark of activated hypothalamus-pituitary-adrenals axis and thus received main attention as a marker of AHF and AKI.

### 2.7. ST2

As a member of the interleukin-1 receptor family, ST2 exists in two different forms: a transmembrane receptor (ST2L) and a soluble decoy receptor (sST2) [36]. It affects the activation of T-helper type 2 (Th2) cells and production of Th2-related cytokines [37]. sST2 has been reported to correlate with cardiovascular events and mortality in patients with AHF and CHF [38–40]. sST2 is significantly related to cardiovascular and all-cause mortality in low-risk populations [41]. sST2 improves prognostic prediction in HF patients with renal dysfunction. Moreover, sST2 is linked to the development of CKD and is associated with cardiovascular events and survival in patients with CKD.

### 2.8. Midregional Proadrenomedullin

Adrenomedullin (ADM), a 52-amino-acid ringed peptide with C-terminal amidation, is a potent vasodilator synthesized in the adrenal medulla, vascular endothelial cells, heart, and elsewhere in response to physical stretch and specific cytokines [42]. Plasma ADM levels are elevated in the heart as a result of pressure and volume overload. It is difficult to measure plasma ADM levels due to its short half-life and existent binding proteins. Midregional proadrenomedullin (MR-proADM) is more stable and is manufactured in a 1 : 1 ratio with active ADM [43]. MR-proADM is a promising biomarker, identifying global disease burden in HF and predicting morbidity in patients with HF [44]. MR-proADM correlates with the development of CKD and predicts the decline of renal function. MR-proADM is associated with cardiovascular and all-cause mortality in patients with renal dysfunction.

### 2.9. Procalcitonin

Procalcitonin is a precursor peptide of hormone calcitonin, and its levels increase particularly following bacterial infection. Procalcitonin has been studied from two perspectives: as a prognostic marker in HF and as a guiding biomarker for adequate therapy [45]. Inflammation is an important pathophysiological factor in HF and may affect prognosis of patients with HF. Patients with HF have significantly higher plasma procalcitonin levels than healthy subjects, and plasma procalcitonin levels are associated with severity of HF. Procalcitonin is associated with mortality and rehospitalization in HF patients with no evidence of infection. Meanwhile, procalcitonin can be applied to guide decision-making with respect to antibiotic therapy in patients with AHF by identifying patients with concomitant or triggering bacterial infection. Procalcitonin-guided antibiotic treatment reduces the duration of antibiotic therapy and improves outcomes in HF patients [46]. Moreover, serum procalcitonin levels are associated with AKI, and elevated procalcitonin levels identify patients at increased risk of AKI [47].

## 3. Renal Biomarkers

### 3.1. Creatinine

As standard indicators of renal function, serum creatinine levels and glomerular filtration rate (GFR) are vital for effective identification of renal diseases and prognostic predication of patients with renal diseases. Both HF and renal diseases play important roles in the occurrence and progression of each other and lead to increased prevalence and mortality of patients with HF and renal diseases. Thus, creatinine can be applied to identify increased prevalence of renal diseases in patients with HF and predict increased mortality in patients with either HF or renal diseases.

### 3.2. Microalbuminuria

Microalbuminuria is related to not only HF and CKD development, but also rehospitalization and mortality rates in the general population [48]. Microalbuminuria predicts prognosis and stratifies risk in patients with HF, independently of GFR and traditional cardiovascular risk factors. However, the mechanisms linking microalbuminuria to HF remain unclear. Microalbuminuria reflects hemodynamic and neuroendocrine interaction of renal tubules and glomerulus with cardiac muscles and vessels and indicates extensive vascular injury caused by cardiovascular risk factors (insulin resistance, blood pressure, glucose, lipids, inflammation, and coagulative abnormality) [51]. Microalbuminuria is a biomarker of endothelial function, vascular permeability, arterial stiffness, and hemodynamic stability in the general population [52]. Angiotensin converting enzyme inhibitor and angiotensin receptor antagonist reduce cardiovascular events and deaths and delay the deterioration of renal function by decreasing microalbuminuria in the general population.

### 3.3. Neutrophil Gelatinase-Associated Lipocalin

Neutrophil gelatinase-associated lipocalin (NGAL) is produced by neutrophils due to an inflammatory reaction. NGAL has elevated levels because of the acute renal tubular injury caused by ischemia and toxicosis. NGAL identifies patients with AKI and serves as a biomarker of AKI. NGAL is a biomarker of CKD and renal transplantation therapy and correlates with residual renal function in patients on dialysis [53]. NGAL predicts worsening renal function, correlates with inflammatory reaction, and mediates ventricular remodeling in AHF [54]. NGAL is produced due to renal injury and inflammatory reactions and serves as a biomarker in parallel with NYHA classification to predict mortality rates in patients with CHF [55].

### 3.4. Cystatin C

As an inhibitor of cysteine protease, cystatin C is initially produced by nuclear cells and is completely filtered and reabsorbed by the kidneys. Cystatin C is not affected by age, sex, race, or muscle volume and can serve as a better biomarker of AKI, CKD, and renal replacement therapy than creatinine [56]. Elevated cystatin C levels suggest the existence of renal dysfunction in patients without CKD based on GFR and microalbuminuria [57]. Cystatin C is related to not only HF progression, but also cardiovascular events and deaths, independently of renal function, in patients with HF [3]. Cystatin C correlates with cardiac function and structure, as well as NT-proBNP, in patients with HF, and is a biomarker of ventricular hypertrophy in patients with hypertension [58].

### 3.5. Kidney Injury Molecule-1

As a transmembrane protein in epithelial cells of renal proximal convoluted tubules, kidney injury molecule-1 (KIM-1) increases because these cells are injured by ischemia or toxicosis [59]. KIM-1 may play protective roles (preventing tubule blocking and inhibiting inflammatory reaction) at the early stage of renal injury and harmful roles (promoting epithelial hyperplasia and aggravating interstitial fibrosis) at its later stage. KIM-1 is a biomarker of renal injury to assess the injury degree and observe the therapeutic effect [54]. KIM-1 identifies patients with AKI or CKD significantly earlier than creatinine, and it predicts prognosis in patients with AKI or CKD. Moreover, KIM-1 identifies the development of AKI or CKD in patients with HF. KIM-1 is elevated in patients with HF and is related to the development of HF. KIM-1 is associated with increased HF, cardiovascular events, and deaths in patients with CKD.

### 3.6. N-Acetyl-*β*-D-Glucosaminidase

As a lysosomal enzyme in the epithelial cells of renal proximal convoluted tubules, N-acetyl-*β*-D-glucosaminidase (NAG) cannot be filtered by the kidneys because of the very large molecular weight [52]. Patients with AKI, CKD, or HF have elevated NAG levels, and NAG is a biomarker that identifies cardiac or renal dysfunction in the general population and patients with urinary tract infection. NAG predicts prognosis in patients with AKI, CKD, or HF [56].

### 3.7. Interleukin-18

Interleukin-18 (IL-18) induces T-lymphocytes and natural killer cells to produce interferon in cell-mediated cytotoxic reaction [60]. IL-18 is a biomarker that identifies patients with AKI and predicts prognosis in these patients [61]. IL-18 may be specific to ischemic kidney injury rather than other kinds of AKI [62]. IL-18 not only has elevated levels and predicts mortality rates but also correlates with vascular stiffness and injury in patients with CKD. IL-18 may be a biomarker of vascular injury and may aggravate myocardial ischemia through mediating inflammation and necrocytosis. IL-18 increases in patients with HF and further increases as HF progresses [63]. IL-18 is related to increased mortality and reduced LVEF in patients with HF. IL-18 predicts cardiovascular prognosis in the general population [64].

### 3.8. Other Biomarkers

Homocysteine and uric acid have deleterious effects on cardiac function and structure and are significantly related to cardiovascular events and mortality [65]. Both of them are affected by renal function and serve as the biomarkers of incident CKD [49]. Meanwhile, patients with reduced urotensin II levels have increased cardiovascular mortality rates, and urotensin II may provide cardiovascular protection and predict cardiovascular deaths in patients with CKD [50]. Urotensin II correlates with ventricular function and vascular atherosclerosis in patients with CKD [66]. Additionally, RAAS plays a significant role in mediating the heart and kidney and promotes the development of CRS [1]. Aldosterone rather than renin is a significant biomarker of incident CKD [49].

## 4. Combination Use

Multimarker strategies may be critical to maximize clinical effects of biomarkers, and they have become increasingly prevalent in diagnosis, therapy, and prognosis of CRS [67]. Combining biomarkers may increase their accuracy, but the optimal combinations need to be defined [68]. The combination of biomarkers indicating hemodynamic stress (BNP) and cardiomyocyte necrosis (cTnT) is more likely to be clinically useful in HF [67]. ST2 provides complementary information to NT-proBNP and cTnT in cardiovascular risk stratification [69]. ST2 in combination with CRP is a valuable tool for identifying patients with HF at risk of death [70]. Combining cardiac and renal biomarkers in multimarker strategies may provide important information on the diagnosis, therapy, and prognosis of CRS [71]. The ideal multimarker strategies would measure relevant biomarkers simultaneously from a single biologic sample and report results in a way that integrates multiple biomarkers into simplified results (such as risk score) that could be applied directly to clinical decision-making [67].

## 5. Strength/Weakness

Comparison of these biomarkers can help in the application and selection of specific biomarkers in different clinical situations. Compared with NT-proBNP and cTnT, cystatin C has an additional ability to assess prognosis and stratify risk in patients with HF [72]. Compared with IL-18 and cystatin C, NGAL has a superior ability to predict the duration of AKI, renal replacement therapy, length of hospital stay, and mortality rates. Cystatin C has the highest specificity in biomarkers of AKI [70]. MR-proADM is superior to NT-proBNP in risk prediction of patients with HF. sST2 and BNP are equally useful for predicting all-cause mortality in patients with AHF. However, compared with BNP, sST2 is not useful as an aid in the diagnosis of AHF [73]. Compared with NT-proBNP, cTnT, and copeptin, MR-proADM is a stronger predictor of cardiovascular deaths in older patients of the emergency department [74]. However, both NT-proBNP and MR-proADM are regarded as equal for diagnosing HF according to European guidelines. It is very significant to note that detailed application and selection of these biomarkers remain a complex process. When these biomarkers aid in diagnosis, therapy, and prognosis of CRS, they must be interpreted within different clinical situations and not solely acted upon [68].

## 6. Future Perspectives

As one of the hottest topics, CRS and its biomarkers have received much attention in recent studies. However, there is still a scarcity of evidence about current biomarkers in CRS and little progress in novel biomarkers in CRS. Moreover, it is very difficult to further classify these biomarkers in each type of CRS due to lacking evidence and considerable overlap. In the future, more studies are needed to support current biomarkers and find novel biomarkers in each type of CRS. Although novel platforms, including genomics, proteomics, and metabolomics, are still under development, they have significant potential to explore biomarkers in CRS [68]. To explore and apply a biomarker in CRS, the following requirements should be considered: (1) identification and classification of CRS, (2) risk stratification and prognostic predication of patients with CRS, and (3) therapeutic guiding and monitoring of these patients [1].

## 7. Conclusion

Biomarkers are not only predictive in HF or renal diseases, but also useful in identifying cardiac dysfunction in renal diseases and renal injury in HF. Thus, they may be applied in order to identify patients with CRS and even assess prognosis and guide therapy in these patients. However, studies on biomarkers have just begun in CRS. Future studies are essential to observe current biomarkers and find novel biomarkers in CRS so as to improve diagnosis, therapy, and prognosis of CRS.

## Figures and Tables

**Table 1:** Biomarkers in cardiorenal syndromes.

Syndromes	Biomarkers	References
Acute cardiorenal (type 1)		
Cardiac	cTnT, BNP, NT-proBNP, MPO, MR-proADM	Ronco et al. [1]; Klip et al. [44]
Renal	Creatinine, cystatin C, NGAL, KIM-1, NAG, IL-18	Ronco et al. [1]
Chronic cardiorenal (type 2)		
Cardiac	BNP, NT-proBNP, CRP, ST2	Ronco et al. [1]; Daniels and Bayes-Genis [38]
Renal	Creatinine, microalbuminuria, cystatin C, CRP, homocysteine, uric acid, urotensin II, aldosterone	Ronco et al. [1]; Iacoviello et al. [48]; Fox et al. [49]; Garoufi et al. [50]
Acute renocardiac (type 3)		
Cardiac	BNP, NT-proBNP	Ronco et al. [1]
Renal	Creatinine, cystatin C, NGAL, KIM-1, NAG, IL-18	Ronco et al. [1]
Chronic renocardiac (type 4)		
Cardiac	BNP, NT-proBNP, CRP	Ronco et al. [1]
Renal	Creatinine, microalbuminuria, cystatin C, homocysteine, uric acid, urotensin II, aldosterone	Ronco et al. [1]; Iacoviello et al. [48]; Fox et al. [49]; Garoufi et al. [50]
Secondary CRS (type 5)		
Cardiac	BNP, CRP, procalcitonin, ST2	Ronco et al. [1]; Daniels and Bayes-Genis [38]
Renal	Creatinine, NGAL, IL-18, KIM-1, NAG	Ronco et al. [1]

BNP: B-type natriuretic peptide; CRP: C-reactive protein; cTnT: cardiac troponin T; IL-18: interleukin-18; KIM-1: kidney injury molecule-1; MPO: myeloperoxidase; MR-proADM: midregional proadrenomedullin; NAG: N-acetyl-*β*-D-glucosaminidase; NGAL: neutrophil gelatinase-associated lipocalin; NT-proBNP: N-terminal proBNP.
